# Factors Associated with the Incidence of Prediabetes in Bogor, Indonesia: A Cohort Study

**DOI:** 10.34172/jrhs.2025.170

**Published:** 2024-12-25

**Authors:** Adzkia Avisena Maghfiroh, Chandrayani Simanjorang, Ulya Qoulan Karima

**Affiliations:** ^1^Public Health Study Program, Faculty of Health Sciences, Pembangunan National University, Jakarta, Indonesia

**Keywords:** Prediabetes, Cohort study, Risk factors, Indonesia, Cumulative incidence

## Abstract

**Background:** Prediabetes is a golden period because the blood sugar levels can be lowered to normal levels, unlike diabetes mellitus. Studies on risk factors of prediabetes in Indonesia have never been conducted before, especially with cohort study design. The purpose of this study was to identify risk factors associated with prediabetes in Indonesia based on a cohort study conducted in Bogor, Indonesia.

**Study Design:** A retrospective cohort study.

**Methods:** This study was conducted using data from the Bogor Cohort Study done by the Ministry of Health of Indonesia, which included individuals aged more than 25 years. Individuals who had impaired fasting glucose (IFG) (100-125 mg/dL) and/or impaired glucose tolerance (IGT) (140-199 mg/dL) at baseline screening were excluded from the study. Demographic characteristics, risk factors, and clinical observations were extracted using a questionnaire. Cox regression was used for data analysis.

**Results:** The cumulative incidence of prediabetes in Bogor was 50.3%. Factors associated with prediabetes were old age (RR: 1.33; 95% CI 1.19, 1.47), female gender (RR: 1.32; 95% CI 1.18, 1.48), overweight (RR: 1.17; 95% CI 1.01, 1.36), obesity (RR: 1.26; 95% CI 1.08, 1.46), central obesity (RR: 1.17; 95% CI 1.02, 1.34), high cholesterol levels (RR: 1.11; 95% CI 1.00, 1.22), and hypertension (RR: 1.27; 95% CI 1.14, 1.42).

**Conclusion:** Age is a dominant risk factor for prediabetes. Therefore, it is important to stick to a healthy lifestyle by doing more physical activities and maintaining a balanced diet since young age to prevent prediabetes.

## Background

 Prediabetes is a condition in which a person has a blood sugar level that exceeds normal limits but cannot be considered a diabetic person. Prediabetes can still be cured and the blood sugar level can be returned to normal range or normoglycemia, unlike diabetes mellitus. If prediabetes is not treated properly, it has the potential to progress to type 2 diabetes mellitus which can eventually cause more severe health complications, such as heart disease, stroke, kidney failure, diabetic retinopathy, and nerve damage.^1^ Individuals with prediabetes are at increased risk of being diagnosed with type 2 diabetes. A study conducted by DeJesus et al in the United States followed a baseline cohort of 10796 patients with prediabetes for 5 years. The results showed that 1845 of them (17.1%) would ultimately develop type 2 diabetes mellitus.^2^ On the other hand, interventions in the form of physical activity in patients with prediabetes have been shown to be effective in reducing blood sugar levels.^3^ Therefore, it is crucial to pay attention to individuals who are in the prediabetes stage in order to prevent bigger health problems.

 Many factors can cause prediabetes. For example, being overweight, being over 45 years old, having a family history of diabetes mellitus, having physical activity less than 3 times a week, having a history of gestational diabetes during pregnancy and giving birth to a baby weighing more than 4 kg, and belonging to a certain race, such as African-American or Hispanic-Latino.^4^ Rooney et al stated that prediabetes can be determined by impaired glucose tolerance (IGT) and/or impaired fasting glucose (IFG). The global prevalence of IGT in 2021 was 9.1% (464 million people). Meanwhile, the global prevalence of IFG in 2021 was 5.8% (298 million people). These two rates are projected to increase to 10% and 6.5% in 2045.^5^ In Indonesia, based on the 2018 Riskesdas data, the prevalence of IFG is 26.3% and the prevalence of IGT is 30.8%.^6^

 Studies on the risk factors for prediabetes in Indonesia, especially with a cohort study design, have never been conducted before. Cohort studies are longitudinal studies that have the most robust scientific evidence in terms of causality among other observational study designs. Hence, the purpose of this study is to identify risk factors associated with prediabetes based on the cohort study conducted in Bogor, Indonesia. The result of this study will be very useful in designing prediabetes and type 2 diabetes mellitus prevention programs. Prevention of prediabetes is of great importance in decreasing the burden of disease, disability-adjusted life years (DALYs), and saving the costs incurred for health facilities.^7^

## Materials and Methods

 This research was a retrospective cohort study. In 2012 and 2015, new samples were included in the study after it was started in 2011. The time span of the data analyzed in this study was from 2011 to 2021; therefore, the follow-up period in this study was approximately 10 years. The population of this study was the residents of Bogor city who were over 25 years old. The population of this research study was all residents of five sub-districts in Central Bogor District, Panaragan, Ciwaringin, Babakan, Babakan Pasar, and Kebon Kalapa.

 The inclusion criteria for this cohort study were willingness to participate and the availability of data. Meanwhile, the exclusion criteria for this study were having prediabetes and/or diabetes mellitus at the time of baseline screening, suffering mental disorders and serious illnesses, being pregnant at the time of baseline screening, passing away, and loss to follow-up. In this study, prediabetes was diagnosed according to the Indonesian Society of Endocrinology (PERKENI) guidelines. Prediabetes was defined as blood sugar levels that exceeded normal limits through blood glucose tests. The diagnosis was confirmed by IFG (100-125 mg/dL) and/or IGT (140-199 mg/dL).^8^

 The sampling technique used in this study was purposive sampling. Individuals who met the inclusion and exclusion criteria were included as the sample of this research. The follow-up of risk factors such as hypertension, blood lipids (cholesterol, high-density lipoprotein [HDL], low-density lipoprotein [LDL], triglyceride), body mass index (BMI), central obesity, smoking, alcohol consumption, mental disorder, and physical activity was carried out 3 times a year at the nearest health service facility (Posbindu), while complete examinations for non-communicable diseases were carried out every 2 years.

 The data collection instrument used in this research was a questionnaire. Behavioral variables and risk factors that were followed through routine follow-ups. Data on physical activity, central obesity, hypertension, total cholesterol, HDL, LDL, triglyceride levels, smoking behavior, alcohol consumption, BMI, and mental disorders were taken from the baseline data. The questionnaire was developed in several stages and was evaluated every year through expert meetings, trials, and workshops. The collection of risk factor surveillance data was carried out using a questionnaire and the WHO STEPwise approach.

###  Statistical analysis

 In this research, multivariable analysis was done by Cox regression model using IBM SPSS version 29.0. Cox regression analysis was used to determine the relationship between the independent variable and the dependent variable by considering the time value. Variables with a *P* value of less than 0.25 in bivariate analysis were eligible for multivariable analysis. The final result will be seen from the Adjusted Relative Risk (RR) value. The most influential or dominant variable was the one that had the highest RR value.

## Results

 Based on the results of the 10-year follow-up of the cohort (2011-2021), the cumulative incidence of prediabetes in Bogor, Indonesia, was 50.3% with an incidence density rate of 573 per person month as indicated in Table 1. In other words, approximately 6 out of 1000 people develop prediabetes every month. Characteristics of the population in this cohort study are presented in Table 2. Participants were divided into 3 age groups: young adults (25-39 years), middle-aged adults (40-59 years), and the elderly (over 60 years). There were 1416 young adults (42.0%), 1772 middle-aged adults (52.6%), and 181 elderly participants (5.4%). History of diabetes mellitus was determined based on the history of diabetes mellitus in parents and/or siblings. In this study, most of the participants had no history of diabetes mellitus (89.2%) while the others (10.8%) had a family history of diabetes mellitus. The number of female participants in this study was 2121 (63.0%) and the number of male participants was 1248 (37.0%). Gestational diabetes was determined by the history of glucose intolerance that was first diagnosed during pregnancy. Most of the participants had no history of gestational diabetes (94.6%), while the others had a history of gestational diabetes (3.9%) or had no records available (1.5%).

**Table 1:** Cumulative incidence of prediabetes in the Bogor Cohort Study

**Variables**	**Number**	**Percent**
Prediabetes		
Yes	1696	50.3
No	1673	49.7
Total	3369	100.0

**Table 2:** Characteristics of the population in the Bogor Cohort Study

**Variables**	**Number**	**Percent**
Age		
Young adult	1416	42.0
Middle-aged adult	1772	52.6
Older adult	181	5.4
Gender		
Male	1248	37.0
Female	2121	63.0
History of diabetes mellitus		
No	3004	89.2
Yes	365	10.8
History of gestational diabetes		
No	3187	94.6
Yes	130	3.9
No record	52	1.5
Education level		
High	1492	44.3
Low	1877	55.7
Income level		
Above average	244	7.2
Below average	3125	92.8
BMI		
Normal	1755	52.7
Severe underweight	79	2.3
Underweight	175	5.2
Overweight	504	15.0
Obesity	836	24.8
Central obesity		
Normal	2122	63.0
Central obesity	1247	37.0
Total cholesterol level		
Normal	1897	56.3
High	1472	43.7
HDL level		
Normal	2319	68.8
Low	1050	31.2
LDL level		
Normal	718	21.3
High	2651	78.7
Triglyceride level		
Normal	2892	85.8
High	477	14.2
Smoking		
No	1524	45.2
Yes	1845	54.8
Alcohol consumption		
No	2606	77.4
Yes	763	22.6
Mental disorder		
No	2514	74.6
Yes	855	25.4
Hypertension		
Normal	2548	75.6
Hypertension	821	24.4
Physical activity		
Enough	2840	84.3
Lack	529	15.7

BMI, Body mass index; HDL, high-density lipoprotein; LDL, low-density lipoprotein.

 The level of education was considered high if the participants had completed high school and/or college or university, and it was considered low when they had no educational background and/or had not completed their education up to junior high school. In this study, 1877 (55.7%) participants had a low level of education and 1492 (44.3%) participants had a high level of education. The level of income was considered high if it exceeded the regional minimum wage in Bogor and low if it did not exceed that limit. More than half of the participants had income levels below the average (92.8%). Only a small portion of them had income levels higher than the regional minimum wage (7.2%).

 Smoking behavior was determined based on the history of smoking in the past few months. In this study, 54.8% of the participants were smokers and 45.2% of them were non-smokers. Alcohol consumption was determined by the history of alcohol consumption in the past few months. Most of the participants did not consume alcohol (77.4%) while the others consumed alcohol (22.6%). BMI was determined based on weight and height measurements during anthropometric measurements. More than half of the participants had a normal BMI (52.7%), followed by obesity (24.8%), overweight (15.0%), underweight (5.2%) and severe underweight (2.3%).

 Physical activity was divided into sufficient ( > 600 metabolic equivalent minutes (MET)/week) and insufficient ( < 600 MET/week). Most participants had sufficient physical activity (84.3%) and the rest of them had insufficient physical activity (15.7%). Central obesity was defined as a waist circumference > 80 cm for women and > 90 cm for men. The majority of the participants had no central obesity (63.0%) while the others had central obesity (37.0%). Individuals were considered to be hypertensive when they had blood pressure ≥ 140/90 mm Hg. Total cholesterol levels were high if they were higher than 200 mg/dL. HDL levels < 45 mg/dL were categorized as low HDL levels and HDL levels > 45 mg/dL were considered as normal HDL levels. LDL levels were considered high when individuals had LDL levels of more than 100 mg/dL and normal if they were less than 100 mg/dL. Triglyceride levels were considered normal when individuals had triglyceride levels ≤ 150 mg/dL and they were considered high if they exceeded it.

 Bivariate analysis was done by Cox regression model and most of the variables were eligible as a candidate for multivariable analysis. Only level of income (*P*=0.46) and mental disorder (*P*=0.96) were ineligible (*P* > 0.25). Multivariable analysis was done using Cox regression model by including all variables in the model and removing them sequentially based on the highest *P* value. This step was continued until no variable had a *P* value above 0.05. Level of education (*P*=0.73), HDL levels (*P*=0.58), LDL levels (*P*=0.44), triglyceride levels (*P*=0.42), alcohol consumption (*P*=0.27), history of diabetes mellitus (*P*=0.25), physical activity (*P*=0.24), smoking (*P*=0.20), and gestational diabetes (*P*=0.17) were not significantly associated with the incidence of prediabetes; therefore, they had to be removed from the model. The final result (fit model) of the multivariable analysis showed that risk factors associated with prediabetes were age, gender, BMI, central obesity, total cholesterol levels, and hypertension. The final model of multivariable analysis is presented in Table 3.

**Table 3:** Fit model of multivariable analysis of the risk factor for prediabetes in the Bogor Cohort Study

**Variables**	**Prediabetes**		**Non-prediabetes**		**Adjusted RR**	***P *** **value**
	**Number**	**Percent**	**Number**	**Percent**		
Age groups						
Young age	605	42.7	811	57.3	Ref.	
Middle age	992	60.0	780	40.0	1.33 (1.19, 1.47)	0.000
Older age	99	52.7	82	45.3	1.22 (0,97, 1.52)	0.084
Gender						
Male	514	41.2	734	58.8	Ref.	
Female	1182	55.7	939	44.3	1.32 (1.18, 1.48)	0.000
BMI						
Normal	788	44.4	987	55.6	Ref.	
Severe underweight	34	43.0	45	57.0	1.06 (0.75, 1.50)	0.729
Underweight	72	41.1	103	58.9	1.01 (0.79, 1.29)	0.918
Overweight	281	55.8	223	44.2	1.17 (1.01, 1.36)	0.035
Obesity	521	62.3	315	37.7	1.26 (1.08, 146)	0.003
Central obesity						
Normal	936	44.1	1186	55.9	Ref.	
Central obesity	760	61.0	487	39.0	1.17 (1.02, 1.34)	0.028
Total cholesterol level						
Normal	879	46.3	1018	53.7	Ref.	
High	817	55.5	655	44.5	1.11 (1.00, 1.22)	0.047
Hypertension						
Normal	1181	46.3	1367	53.7	Ref.	
Hypertension	515	62.7	306	37.3	1.27 (1.14, 1.42)	0.000

BMI, Body mass index; RR. relative risk.

 Middle-aged adults (40-59 years) had 1.3 times higher risk of developing prediabetes compared to other age groups (95% CI 1.19, 1.47;* P=*0.00). Older age was not significantly associated with the incidence of prediabetes (*P*=0.08). There was a significant relationship between gender and prediabetes (*P*=0.000). Females were 1.3 times at higher risk of developing prediabetes than males (95% CI 1.18, 1.48). Individuals who had a BMI above normal were at higher risk of developing prediabetes; in other words, obese people had a 1.3 times higher risk (95% CI 1.08, 1.46; *P*=0.00) and overweight people had a 1.2 times higher risk (95% CI: 1.01, 1.36; *P*=0.03). Meanwhile, the relationship was not significant for severe underweight (*P*=0.79) and underweight (*P*=0.91) categories. Individuals with central obesity had a 1.2 times higher risk of developing prediabetes compared to those without central obesity (95% CI: 1.02, 1.34). There was a significant relationship between central obesity and the incidence of prediabetes (*P*=0.02).

 Total cholesterol levels had a significant relationship with the incidence of prediabetes (*P*=0.04). Individuals with high total cholesterol levels had a 1.1 times higher risk of developing prediabetes compared to individuals with normal cholesterol levels (95% CI: 1.00, 1.22). There was a significant relationship between hypertension and the incidence of prediabetes (*P*=0.00). Based on the results of multivariable analysis, individuals with hypertension had a 1.3 times higher risk of developing prediabetes compared to individuals without hypertension (95% CI: 1.14, 1.42).

## Discussion

 In a study done by Ampeire et al, middle-aged adults had a higher risk of prediabetes compared to other age groups (AOR: 7.4; 95% CI 1.61, 34.29).^9^ Nowadays, the prevalence rates of prediabetes and type 2 diabetes mellitus are increasing in middle-aged adults. A study conducted in Türkiye concluded that prediabetes can start from the age of 20 years. This finding was confirmed by research conducted in 2018 by Aldossari et al in which the incidence of prediabetes was reported in young and middle-aged adults.^10^ Several factors can cause this to happen, including changes in eating patterns, especially when people consume more food and drinks with higher sugar levels accompanied by low physical activity.^11^ Moreover, age is related to the incidence of prediabetes and type 2 diabetes mellitus because most organs show a decrease in function with age. This includes the pancreas which produces insulin and can cause an increase in blood sugar levels.^12^

 A study conducted in Saudi Arabia found that individuals with a BMI above normal (overweight) had a 1.8 times higher risk of developing prediabetes (95% CI: 1.11, 3.06) compared to individuals who had a normal BMI. Furthermore, obese people had a 3.7 times higher risk of developing prediabetes compared to normal BMI (95% CI: 2.30, 6.02).^13^ An increase in BMI can increase the risk of insulin resistance, which can result in prediabetes and type 2 diabetes mellitus.^14^ Moreover, there was a positive association between BMI status and progression of prediabetes to type 2 diabetes mellitus. Individuals with overweight BMI status had a 9.9% lower probability of reversion to normoglycemia compared to normal BMI. In obese people, the probability was lower. Individuals with obesity had a 16.9% lower probability of reversion to normoglycemia than individuals with a normal BMI.^15^ Therefore, controlling BMI is an important factor to prevent prediabetes as well as type 2 diabetes mellitus.

 Central obesity is one of the factors that are associated with the incidence of prediabetes. In a previous study, individuals with central obesity had a 1.7 times higher risk of prediabetes (95% CI: 1.34, 2.24) compared to individuals without central obesity.^16^ A cohort study conducted in China concluded that people with a waist circumference exceeding the threshold had a 1.1 times higher risk of developing prediabetes compared to individuals with a normal waist circumference (95% CI: 1.01, 1.05). Glucose utilization in peripheral tissues and the liver is influenced by changes in tissue proportions and an increase in free fatty acids. This causes gluconeogenesis which results in insulin resistance, a predisposing factor for prediabetes.^17^ This finding was confirmed by another study reporting that people with central obesity are more likely to suffer from type 2 diabetes mellitus and also are at a higher risk of developing cardiovascular disease. The adoption of sedentary a lifestyle could be the reason. Sedentary lifestyle is positively associated with abdominal obesity, but it can negatively affect insulin sensitivity. Therefore, it can increase the risk of diabetes mellitus.^18^

 Yan et al in 2023 stated that women were 1.2 times at a higher risk of prediabetes than men (95% CI: 1.02, 1.61).^14^ Differences in body composition of women and men make women tend to have a higher risk of developing prediabetes and type 2 diabetes mellitus. Women have more abdominal fat and perivisceral adiposity compared to men. Therefore, women have a higher risk of prediabetes and type 2 diabetes mellitus as well as cardiovascular diseases.^19^ Women with prediabetes are also at higher risk of experiencing progression to type 2 diabetes mellitus.^20^ The existence of brown adipose tissue (BAT) in men and women is negatively related to the incidence of prediabetes by reducing the probability of insulin resistance and increasing adiponectin. Yet, there are differences in the distribution of this tissue between males and females. Additionally, the divergence between men and women in environmental factors, lifestyle, disease treatment, nutrition, socioeconomic status, and levels of stress can also influence the risk of developing prediabetes.^21^

 Individuals with high total cholesterol levels have a higher risk of developing prediabetes. On the other hand, individuals with prediabetes have higher total cholesterol levels compared to individuals without prediabetes.^22^ Elyantari et al reported that individuals who had a high level of total cholesterol, had a 3 times higher risk of developing prediabetes compared to individuals who had normal cholesterol levels (95% CI 1.44, 6.54). Increased cholesterol levels in the blood can be an early sign of pancreatic beta cell dysfunction, which may influence the secretion of insulin. This condition can ultimately cause hyperglycemia.^23^

 A study conducted in Zhejiang, China, found that individuals with hypertension had a 2.6 times higher risk (95% CI: 2.06, 3.20) of developing prediabetes compared to individuals without hypertension (*P*=0.00).^16^ Furthermore, individuals with hypertension had a 3.2 times higher risk of developing prediabetes compared to individuals without hypertension (95% CI 1.45, 7.31). Hypertension is also one of the main risk factors for glucose intolerance and diabetes complications. High blood pressure is associated with low insulin sensitivity, which causes disruption of glucose absorption in the body. High insulin levels can increase sodium retention in the kidney tubules, which can cause hypertension. Therefore, hypertension and diabetes are related to each other.^24^ In addition, hypertension is oftentimes found in individuals with prediabetes.^25^ There is also a relationship between prediabetes and the risk of mortality or cardiovascular disease. Yet, the relationship is strong only when prediabetes is combined with other metabolic syndromes such as hypertension.^26^

**Highlights**

- The cumulative incidence of prediabetes in Bogor is 50.3%.
- The incidence density rate of prediabetes is 573 per 1000 person-month.
- Factors associated with the incidence of prediabetes in Bogor are age, gender, body mass index (BMI), central obesity, total cholesterol, and hypertension.

## Conclusion

 Our study found that middle-aged adults are more likely to develop prediabetes than young adults and the elderly. Due to differences in body composition, women are more likely to develop prediabetes than men. People with overweight and obesity are at higher risk of developing prediabetes than people with other BMI categories since these people have a higher probability of experiencing insulin resistance, which is a predisposing factor for prediabetes. Individuals who have central obesity are at higher risk of developing prediabetes since they are also at higher risk of insulin resistance. High cholesterol levels and hypertension are found to be more prevalent in individuals with prediabetes and vice versa.

## Acknowledgments

 The authors would like to thank Badan Kebijakan Pembangunan Kesehatan (BKPK), the Ministry of Health of Indonesia for the availability of data and the permission to use them in this research.

## Competing Interests

 The authors have no conflict of interests.

## Ethical Approval

 Researchers guarantee the confidentiality of the identity and data of respondents. The information obtained will be used for research purposes. This research was conducted after obtaining approval from the UPN Veteran Jakarta Health Research Ethics Commission (KEPK) which was issued on May 17, 2024, with letter number 222/V/KEP/2024.

## Funding

 No funding was received for this research.
